# The Effect of Drying Methods on the Pore Structure of Balsa Wood Aerogels

**DOI:** 10.3390/polym17121686

**Published:** 2025-06-17

**Authors:** Min Yin, Zongying Fu, Xia Yu, Ximing Wang, Yun Lu

**Affiliations:** 1 College of Material Science and Art Design, Inner Mongolia Agricultural University, Hohhot 010018, China; ym1872@emails.imau.edu.cn (M.Y.); xyu02032023@163.com (X.Y.); 2 Key Laboratory of Wood Science and Technology of National Forestry and Grassland Administration, Research Institute of Wood Industry, Chinese Academy of Forestry, Beijing 100091, China; zyfu@caf.ac.cn

**Keywords:** wood aerogel, drying methods, pore structure, thermal conductivity

## Abstract

Drying constitutes an essential step in aerogel fabrication, where the drying method directly determines the pore structure and consequently influences the material’s functionality. This study employed various drying techniques to prepare balsa-wood-derived aerogels, systematically investigating their effects on microstructure, density, and performance characteristics. The results demonstrate that different drying methods regulate aerogels through distinct pore structure modifications. Supercritical CO_2_ drying optimally preserves the native wood microstructure, yielding aerogels with superior thermal insulation performance. Freeze-drying induces the formation of ice crystals, which reconstructs the microstructure, resulting in aerogels with minimal density, significantly enhanced permeability, and exceptional cyclic water absorption capacity. Vacuum drying, oven drying, and natural drying all lead to significant deformation of the aerogel pore structure. Among them, oven drying increases the pore quantity of aerogels through volumetric contraction, thereby achieving the highest specific surface area. However, aerogels prepared by air drying have the highest density and the poorest thermal insulation performance. This study demonstrates that precise control of liquid surface tension during drying can effectively regulate both the pore architecture and functional performance of wood-derived aerogels. The findings offer fundamental insights into tailoring aerogel properties through optimized drying processes, providing valuable guidance for material design and application development.

## 1. Introduction

Wood-derived aerogels, as a renewable, biodegradable, and environmentally friendly porous material, possess an inherent advantage in addressing environmental pollution. Characterized by high surface area, high porosity, and hierarchical porous structures [1], these aerogels demonstrate exceptional performance in environmental remediation. The high porosity contributes to substantial specific surface area, which in turn provides abundant active sites for pollutant adsorption. These aerogels exhibit outstanding adsorption capabilities for heavy metal ions [2], such as copper, uranium, and cesium ions [3,4,5], making them suitable for wastewater treatment. They effectively adsorb non-metallic elements such as phosphorus and can also separate microplastics from aqueous systems [6,7,8,9]. Their superior crude oil adsorption performance [10,11] and capacity to remove biological contaminants like tetracycline from the environment have been well documented [12]. Beyond aqueous applications, wood aerogel composites show promise for airborne particulate matter removal [13] and carbon dioxide capture [14]. The material’s multifunctionality extends to thermal insulation, sound absorption, seawater desalination, radiative cooling, energy storage, and photothermal conversion [15,16,17,18]. With advancements in modification technologies and increasing environmental requirements, wood-derived aerogels are emerging as a foundational platform for green functional materials. In-depth research on sustainable wood aerogels holds significant importance for developing next-generation environmental solutions. Their unique combination of eco-friendly characteristics and versatile performance positions them as promising candidates for addressing multiple environmental challenges.

Wood-derived aerogels consist of solid lignocellulosic components and intercellular pores, with their performance closely related to pore structure. The properties of porous materials are significantly affected by both average pore diameter and size distribution [19]. Clara et al. successfully reduced the thermal conductivity from 0.024–0.028 Wm^−1^K^−1^ to 0.018–0.021 Wm^−1^K^−1^ by decreasing the aerogel pore size [20]. Chen et al. fabricated aerogels with anisotropic pore structures through directional freezing in ethanol at −30 °C, creating honeycomb-like porous structures in the transverse direction and well-aligned tunnel structures longitudinally, which exhibited excellent self-supporting and compression-resistant capabilities. Compared to ethanol-based directional freezing, liquid nitrogen directional freezing produced aerogels with smaller pores, resulting in slower ink transport and transfer rates [21]. Li et al. demonstrated that water transport rate in aerogel evaporators was pore-size dependent, increasing from 31.94 to 75.84 g min^−1^ as pore size grew from 21.6 μm to 91.9 μm. Optimal solar evaporation performance (2.86 kg m^−2^ h^−1^) was achieved at 73.4 μm pore size [22]. Ruan et al. prepared cellulose nanocrystal aerogels showing superior acoustic absorption in low-to-medium frequencies (600–3000 Hz) when processed by liquid nitrogen directional freezing, which created smaller pores and lower bulk density compared to conventional freeze-drying [23]. Wu et al. developed aerogel/wood composites via freeze-drying, combining wood’s parallel channels with aerogel’s porous structure to create unique disordered/ordered pore architectures. This design, coupled with super wettability and gravity effects, achieved exceptional oil-in-water emulsion separation efficiency (99.25%) and high flux (2580 L·m^−2^·h^−1^) [24]. Yu et al.’s wood composite aerogels featured vertical microchannels and abundant nanopores, enabling remarkable thermal insulation with nighttime temperatures significantly above ambient conditions [25]. These studies collectively demonstrate that pore structure directly determines wood aerogel performance, suggesting that precise pore structure engineering can effectively tailor aerogel properties for specific applications.

The pore structure of wood-based aerogels is constructed during the drying process, with the drying method directly influencing the properties of the aerogel. Currently, the most commonly employed drying techniques include freeze-drying, supercritical CO_2_ drying, and ambient drying [26,27]. Guan et al. fabricated wood-based aerogels using balsa wood through freeze-drying, creating wave-like stacked layers within the aerogel structure. This configuration endowed the aerogel with high mechanical compressibility, elastic recovery rate, and excellent oil/water absorption selectivity, achieving an oil absorption capacity as high as 41 g g^−1^ [28]. Deeptanshu et al. prepared mesoporous-dominant aerogels via supercritical CO_2_ drying of TEMPO-oxidized wood cellulose. The resulting 10 mm thick aerogels reached up to 80% light transmittance, which decreased with increasing thickness. At a density of 67 mg/cm^3^, the aerogel’s thermal conductivity was reduced to 18.5 mW/m·K [29]. Mao et al. developed composite nanocellulose fiber aerogels using ambient pressure drying. These aerogels exhibited varying pore structures and sizes across different layers, along with superior electromagnetic interference shielding efficiency and ultralow reflection coefficients in the 1–18 GHz frequency band [30]. While different drying methods impart distinct properties to aerogels and directly affect their pore structure and size, the specific mechanisms by which the drying process influences aerogel microstructure require further in-depth investigation.

**Figure 1:**
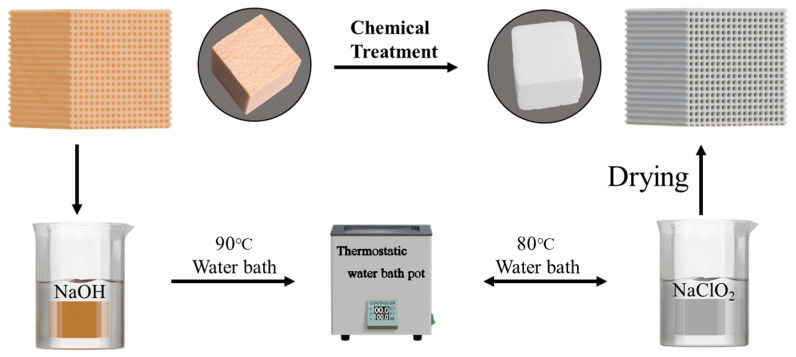
Schematic diagram of wood aerogel preparation.

Therefore, this study successfully prepared five types of wood aerogels using balsa wood (BW) as the base material: freeze-dried wood aerogel (FD-A), supercritical CO_2_-dried wood aerogel (SCD-A), vacuum-dried wood aerogel (VD-A), oven-dried wood aerogel (OD-A), and naturally dried wood aerogel (ND-A), the preparation process is shown in Figure 1. The aim was to investigate the effects of different drying methods on the microstructure (SEM), specific surface area, pore size, and other parameters of wood aerogels. Understanding the influence of drying processes on the pore structure of aerogels will contribute to the improvement of aerogel preparation techniques and performance optimization. The research findings will deepen our comprehension of the structure–property relationships in porous materials and provide a theoretical basis for material design. Furthermore, this study will provide theoretical support for promoting the green manufacturing and industrial applications of biomass-based aerogels.

## 2. Materials and Methods

### 2.1. Materials

The balsa wood was pre-cut into dimensions of 15 mm (length) × 15 mm (width) × 15 mm (height) and provided by Guangdong Yihua Life Co., Ltd., Shantou, China. Absolute ethanol (analytical grade) was supplied by Tianjin Fuyu Fine Chemical Co., Ltd., Tianjin, China. Sodium chlorite (NaClO_2_) (analytical grade) was from Shanghai Chuangcai Technology Co., Ltd., Shanghai, China. Glacial acetic acid (purity grade) was provided by Tianjin Kemiao Chemical Reagent Co., Ltd., Tianjin, China. Sodium hydroxide (NaOH) (analytical grade) was purchased from Tianjin Fuchen Chemical Reagent Factory, Tianjin, China. Distilled water was prepared using a laboratory-grade pure water system, model DL-P3-20TJ, from Anhui Zhongke Duling Commercial Electrical Co., Ltd., Hefei, China.

### 2.2. Preparation of Wood Aerogels

The experimental material selected was balsa wood, from which the hemicellulose and lignin were removed using a potassium hydroxide–sodium chlorite method. The concentration of NaOH was 2 wt%, with a water bath temperature of 90 °C, and the treatment time was 7 h for hemicellulose removal. The NaClO_2_ concentration was 1 wt%, and the pH was adjusted to 4.5–4.6 using glacial acetic acid. The water bath temperature was maintained at 80 °C, with a total processing time of 18 h. The NaClO_2_ solution was replaced every 9 h until the wood samples attained complete whitening. The sample was washed with distilled water and set aside.

### 2.3. Freeze Drying to Prepare Wood Aerogel

The equipment used was a freeze dryer (model SCIENTA-10N/D) produced by Ningbo Xinzhi Biotechnology Co., Ltd., Ningbo, China. The lignin-removed wood hydrogel was placed in a −70 °C freezer to solidify, then transferred to the freeze-drying equipment. The drying process was conducted at a temperature of −54.9 °C under a vacuum of 1 Pa until completion.

### 2.4. Solvent Exchange

The equipment used was a peristaltic pump (model BT101S) produced by Baoding Longer Precision Pump Co., Ltd., Baoding, China. At room temperature, the wood with removed hemicellulose and lignin was placed in an anhydrous ethanol solution. The peristaltic pump was used to circulate the ethanol, and the ethanol solution was replaced every 6 h until the exchange was complete.

### 2.5. Supercritical Carbon Dioxide Drying to Prepare Wood Aerogel

The equipment used was a supercritical dryer (model SCD-350M) produced by Shianjia Beijing Biotechnology Co., Ltd., Beijing, China. The completed sample was placed inside the supercritical instrument. The inlet pressure was increased at a rate of 0.01 MPa/min until the pressure in the supercritical instrument matched that of the gas cylinder. The inlet valve was then fully opened, and the system was cooled for 30 min. The vent valve was opened to release gas at a rate of 1 L/min. These steps were repeated multiple times until no liquid was discharged, after which the vent valve was closed. The temperature was then raised to 35 °C, and the pressure was maintained at 8 MPa for 1 h before the gas was discharged.

### 2.6. Oven Drying to Prepare Wood Aerogel

The equipment used was a drying oven (model UN110) produced by Memmert GmbH, Schwabach, Germany. The processed sample was placed in the drying oven and dried at a temperature of 40 °C until the mass no longer changed.

### 2.7. Vacuum Drying to Prepare Wood Aerogel

The vacuum drying process was performed using a DZF-6030B vacuum drying oven (Shanghai Yiheng Scientific Instrument Co., Ltd., Shanghai, China). Following solvent exchange, the samples were placed in the vacuum chamber and dried at 40 °C under a reduced pressure of −50 kPa (gauge pressure, corresponding to 51.3 kPa absolute pressure) until the mass no longer changed.

### 2.8. Nature Drying to Prepare Wood Aerogel

Taking advantage of the volatile nature of anhydrous ethanol at room temperature and atmospheric pressure, the processed specimens were placed in Petri dishes and kept in an indoor environment until the mass no longer changed.

### 2.9. Characterization of Scanning Electron Microscopy (SEM)

The equipment model used is the Czech TESCAN MIRA LMS, Tescan Trading (Shanghai) Co., Ltd., Shanghai, China. The sputter coating target material is platinum. The bulk sample is directly adhered to conductive tape and then coated with gold for 45 s using the Quorum SC7620 sputter coater, Nanjing Tansi Technology Co., Ltd., Nanjing, China, with a gold sputtering current of 10 mA. Afterward, the TESCAN MIRA LMS scanning electron microscope is used to capture the sample morphology, with an acceleration voltage of 3 kV and the SE2 secondary electron detector.

### 2.10. Crystallinity Testing

For the crystallinity analysis, a Bruker D2 Phaser X-ray diffractometer (XRD) from Bruker Corporation, Karlsruhe, Germany was used. Samples of balsa wood and wood-derived aerogel sheets, with dimensions of 15 mm (length) × 15 mm (width) × 1 mm (thickness), were prepared for testing. The aerogel sheets were derived from freeze-dried aerogels.

### 2.11. Characterization of Density

The equipment used was an electronic balance (model CN-LQC20002) produced by Kunshan Youkeweit Electronic Technology Co., Ltd., Kunshan, China. The mass of the aerogel was measured using the balance. For each drying method, three independent aerogel samples were prepared, with each sample undergoing triplicate dimensional and mass measurements, and the results were averaged. The sample density was then calculated based on the mass and volume of the wood aerogel. The calculation formula is shown in Equation (1).

(1) $$\rho =\frac{M}{V_{0}}$$

where ρ is the wood aerogel density in g/cm^3^, *M* is the mass of the wood aerogel in g, and *V*_0_ is the volume of the wood aerogel in cm^3^.

### 2.12. Characterization of Nitrogen Adsorption–Desorption

The equipment used was a fully automated specific surface area and porosity analyzer (model Quantachrome Autosorb IQ) produced by Quantachrome Instruments, Boynton Beach, FL, USA. Cubic samples measuring 3 (width) × 3 (height) × 15 (length) mm were degassed at 100 °C, followed by nitrogen adsorption–desorption testing at 77 K. Based on the N2 adsorption isotherms and by applying the BET and BJH equations, the specific surface area and pore size distribution of the samples were calculated.

### 2.13. Characterization of Thermal Conductivity

The equipment used included an infrared thermal imager (model PC210) produced by Guide Company (Wuhan Guide Infrared Co., Ltd., Wuhan, China.) and an electric heating plate (model DB-2EES) produced by Bangxi Instrument Technology (Shanghai) Co., Ltd., Shanghai, China. The aerogel and balsa wood samples were weighed three times each, with the average masses recorded as follows: 0.26 g (FD-A), 0.27 g (SCD-A), 0.27 g (VD-A), 0.23 g (OD-A), 0.27 g (ND-A), and 0.48 g (BW). The wood aerogel and balsa wood samples, each with a height of 15 mm, were placed vertically along their axial direction on a heating plate. An infrared thermal imaging camera was then used to capture temperature changes on both the axial and cross-sectional top surfaces, and the infrared thermal imaging camera (Wuhan Guide Infrared Co., Ltd., Wuhan, China.) was used to capture temperature changes in both axial and cross-sectional directions. The heating plate was set to 120 °C, and after the temperature stabilized, the wood aerogel was placed on the plate. Temperature changes were measured every 10 s, with a total testing time of 140 s.

### 2.14. Characterization of Water Absorption Performance

Three replicate samples were set up to evaluate the effect of immersion time in methylene blue (MB) solution (weighing every 30 s, with a total test time of 360 s) on the water absorption performance of the wood aerogel. During the test, the wood aerogels were immersed in a 0.01% MB solution. The weight of each wood aerogel was recorded before and after immersion. The water absorption rate, Y (%), of the wood aerogel can be calculated using the following formula:

(2) $$Y=\frac{Z-M}{M}\times 100\%$$

where *Z* is the weight of the wood aerogel after immersion in the MB solution, and M is the weight of the wood aerogel before immersion in the MB solution. The average value is calculated from three identical samples.

### 2.15. Cyclic Compression Performance

The mechanical properties related to radial/tangential compression were evaluated using a universal testing machine (KXWW-01C, Chengde Kebiao Testing Instrument Manufacturing Co., Ltd., Chengde, China) equipped with a 100 N force sensor. The loading and unloading speeds were maintained at a constant 5 mm/min. The wood aerogel samples had dimensions of 15 mm (length) × 15 mm (width) × 15 mm (height). The compression thickness was 7.5 mm, corresponding to a compressive strain of 50%. The number of cycles was 30. The dimensions of the aerogel were recorded before and after the test.

### 2.16. Characterization of Cyclic Water Absorption Performance

At room temperature, the reusability of the aerogel was evaluated by measuring the water absorption performance of three aerogel samples after undergoing 23 fixed absorption–compression cycles. Each square aerogel sample with a side length of 15 mm was first fully immersed in an MB solution for 3 min, then removed, and its weight was recorded. Subsequently, a steel ruler was used to compress the aerogel to half its thickness, repeating the compression 3 to 5 times until no more water was extruded, after which the weight was recorded again. The water absorption rate of the wood aerogel was calculated using the aforementioned formula in Equation (2). The next immersion–compression cycle begins by re-submerging the wood aerogel in water until it is saturated, then removing it and repeating the remaining steps until 23 cycles are completed. The average value is calculated from three identical samples.

## 3. Results and Discussion

### 3.1. Wood Aerogel Drying Rate

**Figure 2:**
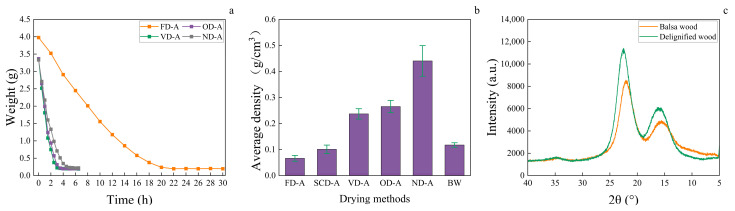
(**a**) Average mass change of wood aerogels during drying process. (**b**) Average density of wood aerogels and balsa wood with error bars. (**c**) XRD of balsa wood and delignified wood.

As shown in Figure 2a, the anhydrous ethanol in the aerogel was removed as the drying process proceeded, leading to a corresponding decrease in mass. The slope of the aerogel mass curve decreases with increasing drying time, indicating a gradual reduction in drying rate as the drying process progresses. During the initial drying stage, a greater amount of solvent is removed from the sample, resulting in a higher drying rate. In the later drying stages, as the solvent mass within the sample diminishes, the drying difficulty increases, leading to a decreased drying rate. The freeze-dried sample (FD-A) exhibited the longest drying duration (22 h) and the lowest drying rate. This extended drying process results from the sublimation mechanism in freeze-drying, where water transitions directly from solid to vapor phase while being removed from the aerogel matrix, combined with the necessity to maintain low-temperature and low-pressure conditions, collectively leading to a reduced drying rate. The naturally dried sample (ND-A) required 5 h for complete drying, longer than the oven-dried samples (OD-A). This extended period results from ND-A’s lower drying temperature, where ambient temperature serves as the primary energy source that directly governs the drying rate. In contrast, vacuum drying (VD-A) exhibited superior drying efficiency (3 h) compared to OD-A (3.5 h) despite identical temperature conditions. This enhancement arises from the reduced intermolecular interactions of ethanol under vacuum, which lowers its boiling point and consequently accelerates the evaporation process. Under conditions of reduced pressure and elevated temperature, the decreased surface tension of ethanol minimizes structural deformation in aerogels, while the lowered boiling point reduces the energy required for liquid-to-vapor phase transition. These combined effects lead to enhanced drying rates under isothermal conditions [31].

### 3.2. Wood Aerogel Density

Density significantly influences the sound absorption [32], thermal conductivity [33], mechanical properties [34], and water absorption performance of aerogels. Under identical mass and volume conditions, higher cellulose content results in reduced pore space proportion and consequently greater wood aerogel density. SEM analysis combined with drying process observations reveals that SCD-A exhibits the minimal density variation before and after drying, as its pore structure remains unaffected by surface tension during supercritical CO_2_ drying. As shown in Figure 2b, the ascending order of wood aerogel density is FD-A < SCD-A < VD-A < OD-A < ND-A, indicating that the drying method directly determines the final density. The crystallinity of the delignified wood increased compared to balsa wood (Figure 2c), indicating the successful removal of lignin and hemicellulose. The lower density of FD-A compared to SCD-A indicates that, at equivalent volume, FD-A aerogels contain less cellulose, attributable to volumetric expansion during freeze-drying as water transforms into ice crystals. In contrast, VD-A, OD-A, and ND-A exhibit higher densities than SCD-A, confirming volume shrinkage during these drying processes. Greater shrinkage corresponds to increased density, as these methods involve surface tension effects from anhydrous ethanol evaporation. Among them, ND-A shows the highest density due to the combined effects of the lowest temperature and highest ambient pressure during natural drying, which maximizes ethanol’s surface tension. The capillary forces induced by surface tension compress the pore structure, modifying the aerogel volume and ultimately altering its density. This mechanism explains the density variations observed across different drying techniques.

### 3.3. Wood Aerogel Microstructure

**Figure 3:**
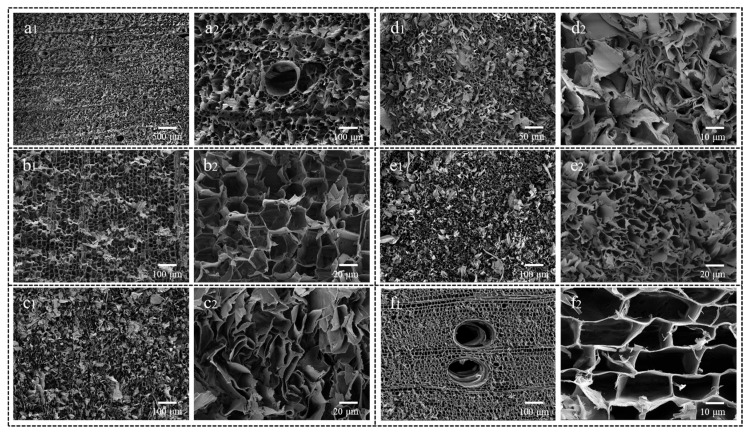
SEM images of the cross-sections of wood aerogels and balsa wood: (**a1**,**a2**) freeze-dried wood aerogel; (**b1**,**b2**) supercritical CO_2_-dried wood aerogel; (**c1**,**c2**) vacuum-dried wood aerogel; (**d1**,**d2**) oven-dried wood aerogel; (**e1**,**e2**) naturally dried wood aerogel; (**f1**,**f2**) balsa wood.

Figure 3 shows that different drying methods have distinct effects on the pore structure of wood aerogels. During freeze-drying, the volume expansion of water upon freezing and the compression of cellulose by ice crystals lead to alterations in both pore size and morphology of the aerogel, as shown in Figure 3(a1,a2). In contrast, supercritical CO_2_ drying requires replacing water in the wood aerogel with anhydrous ethanol prior to drying, a process that does not affect pore structure. Since supercritical CO_2_ exhibits no surface tension, the drying process preserves the original pore structure derived from balsa wood with minimal changes, as shown in Figure 3(b1,b2). For VD-A, OD-A, and ND-A samples, although water is similarly replaced with anhydrous ethanol before drying, the inevitable surface tension of ethanol during drying generates capillary forces that modify the wood’s pore structure during ethanol removal. Among these, ND-A shows the most significant structural changes and greatest volume shrinkage (Figure 3(e1,e2)). This occurs because liquid surface tension is temperature-dependent. Lower environmental temperatures reduce molecular thermal motion, thereby increasing surface tension and, consequently, capillary forces. This results in greater shrinkage of the aerogel [35]. VD-A and OD-A dried at identical temperatures experience similar ethanol surface tension and consequently comparable capillary forces during drying, both being lower than ND-A. Thus, their volume shrinkage is less pronounced than ND-A. Reduced environmental pressure further decreases shrinkage rate because lower pressure weakens intermolecular forces at the liquid surface, thereby reducing surface tension. This explains why vacuum drying results in less shrinkage than oven drying [36]. A comprehensive analysis of Figure 2a reveals that both drying temperature and ambient pressure significantly influence the pore structure formation and drying rate of wood aerogels through their effects on the surface tension and boiling point characteristics of anhydrous ethanol.

After lignin removal from between the wood cells, the resulting spaces become filled with water molecules. During freezing, ice crystal formation in the aerogel causes random changes in wood cell wall morphology. SCD-A best preserves the original wood microstructure, though with noticeably thinner cell walls compared to balsa wood due to hemicellulose and lignin removal. In VD-A, OD-A, and ND-A samples, the inter-cell-wall spacing is significantly reduced compared to balsa wood. Capillary forces during drying cause cell walls to converge inward from both sides, with the degree of deformation influenced by anhydrous ethanol surface tension. Some pore structures disappear due to this deformation [37].

### 3.4. The Water Absorption Performance of Wood Aerogels

**Figure 4:**
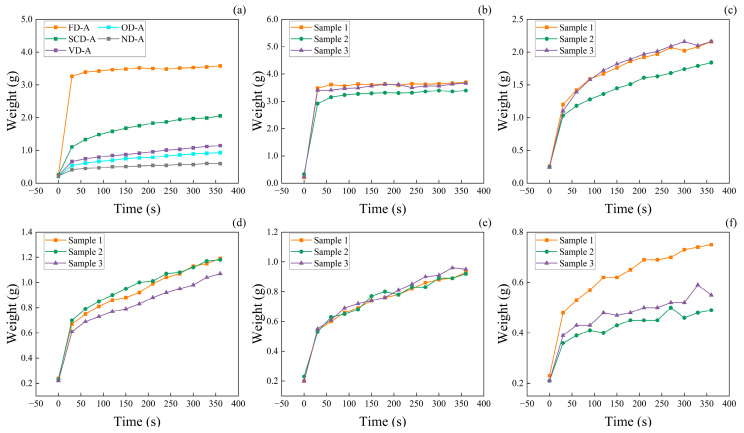
Change in water absorption mass of wood aerogels over time: (**a**) average water absorption mass; (**b**) freeze-dried wood aerogel; (**c**) supercritical CO_2_-dried wood aerogel; (**d**) vacuum-dried wood aerogel; (**e**) oven-dried wood aerogel; (**f**) naturally dried wood aerogel.

The water absorption performance of wood-based aerogels is influenced by factors such as density, pore size, pore volume, and pore connectivity [38,39,40,41]. As analyzed from Figure 4, the average water absorption rate and capacity of the aerogels are affected by the drying method, with the initial average masses being 0.26 g (FD-A), 0.25 g (SCD-A), 0.23 g (VD-A), 0.21 g (OD-A), and 0.22 g (ND-A), respectively. FD-A demonstrates the highest absorption rate and capacity, while ND-A has the lowest, as shown in Figure 4a. Combining the analysis of Figure 2b, it is evident that the average water absorption capacity and rate are inversely proportional to density—higher density leading to lower absorption capacity and rate. Further analysis of Figure 3 demonstrates that FD-A, compared to other drying methods, undergoes distinct pore structure modifications induced by ice crystal formation. These modifications significantly enhance pore interconnectivity. Coupled with its characteristically low density and high porosity, this unique structural configuration results in superior water absorption capacity and absorption rate [42]. In contrast, SCD-A shows minimal changes in pore structure before and after drying. However, VD-A, OD-A, and ND-A all experience pore shrinkage during drying due to capillary forces. The extent of shrinkage increases with the rising surface tension of anhydrous ethanol. This suggests that pore size and porosity are critical factors influencing the absorption performance. Specifically, higher porosity and larger pore size lead to greater absorption capacity and faster absorption rate. As evidenced by Figure 3, the vessels preserved in the aerogels originate from the balsa wood, and these retained vessel structures provide preferential pathways for capillary-driven water penetration.

### 3.5. Thermal Insulation Properties of Wood Aerogels

**Figure 5:**
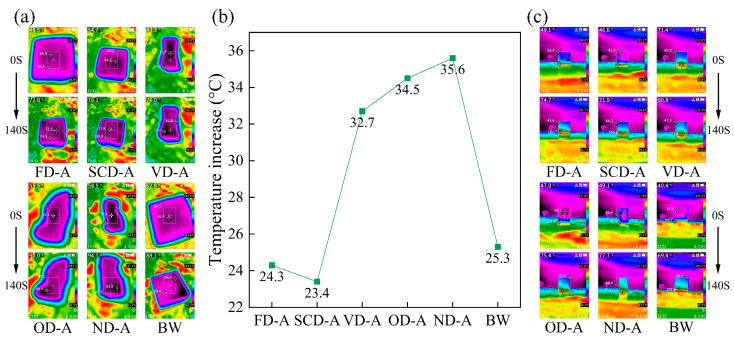
Wood aerogel heating at 120 °C: (**a**) top heating; (**b**) wood aerogel and balsa wood temperature increase; (**c**) axial heating.

Under identical temperature conditions, the thermal insulation performance of aerogels is influenced by factors such as density, pore size, and porosity [27,29,42], while pore connectivity also affects their insulating properties [43]. As shown in Figure 5, wood aerogels prepared by different drying methods exhibit variations in axial thermal insulation performance for samples of the same height. The top surface temperature of balsa wood and SCD-A increased by 25.3 °C and 23.4 °C, respectively. This is attributed to the minimal changes in porosity and pore size of CSD-A before and after drying. The removal of lignin and hemicellulose effectively reduced the density of CSD-A while also endowing the wood aerogel with a more porous structure. ND-A exhibited the highest temperature rise of 35.6 °C at the top surface, while FD-A showed a temperature increase of 24.3 °C, which was higher than that of SCD-A. This phenomenon occurs because ice crystal formation during freeze-drying enlarges the pore size of the wood aerogel, and ice crystal growth enhances pore permeability. Combined with Figure 4a, it is evident that freeze-drying improves the water absorption capacity of the wood aerogels but compromises their thermal insulation performance. The top surface temperature rises of VD-A, OD-A, and ND-A increased sequentially. When correlated with Figure 2b and Figure 3, their density and degree of deformation also showed a progressive increase. This indicates that the surface tension of anhydrous ethanol reduces the pore size of wood aerogels while increasing density, ultimately leading to diminished thermal insulation performance. Furthermore, it demonstrates that the surface tension of anhydrous ethanol modifies the pore structure of wood aerogels, thereby altering both density and thermal barrier effects. Different drying processes significantly affect the microstructural characteristics of aerogels. As the drying method varies, corresponding changes occur in the pore structure and density of the aerogel. When the aerogel density increases under identical volume conditions, its porosity correspondingly decreases, pore size reduces, and the material becomes more compact. These structural alterations directly impact the thermal properties: under the same temperature conditions, aerogels with higher density exhibit more pronounced temperature increases due to enhanced solid-phase thermal conduction, thereby deteriorating their thermal insulation performance. It is feasible to regulate the surface tension of anhydrous ethanol through temperature and environmental pressure adjustments to modify the pore structure of wood aerogels for performance optimization. From the axial infrared images, it can be observed that the heat transfer in wood aerogels occurs from bottom to top, indicating that the heat conduction direction of the wood aerogel is primarily governed by the axial alignment of cellulose.

### 3.6. Wood Aerogel Nitrogen Adsorption

**Figure 6:**
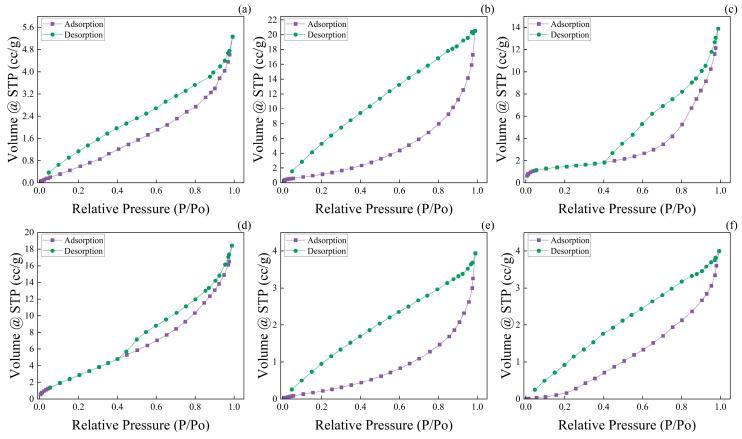
Nitrogen adsorption–desorption isotherms of wood aerogels and balsa wood. (**a**) Freeze-dried wood aerogel. (**b**) Supercritical CO_2_-dried wood aerogel. (**c**) Vacuum-dried wood aerogel. (**d**) Oven-dried wood aerogel. (**e**) Naturally dried wood aerogel. (**f**) Balsa wood.

During the drying process, the volume of wood aerogel shrinks, and its density increases. The volume shrinkage of the wood aerogel results from a reduction in the pore size. Throughout this process, the pore size decreases, while the number of pores may remain unchanged. As shown in Figure 6, the nitrogen adsorption isotherms of aerogels prepared using five drying methods, along with balsa wood, all exhibit characteristic Type IV isotherms. In the low-pressure region, the isotherm curves of the aerogels deviate toward the *Y*-axis, whereas that of balsa wood deviates toward the *X*-axis. The nitrogen adsorption capacity of SCD-A in the medium-pressure region was significantly higher than that of balsa wood, while OD-A exhibited a further increase in adsorption capacity compared to SCD-A, indicating that OD-A possesses enhanced nitrogen adsorption capability. Notably, although SCD-A and OD-A showed similar mesopore sizes (Table 1 BJH), OD-A demonstrated a markedly higher density than SCD-A (Figure 2b). SEM observations in Figure 3 revealed that while OD-A underwent volume shrinkage during drying, its pore structure remained intact. This suggests that under equivalent volume conditions, OD-A contains a greater quantity of mesopores. In contrast, both FD-A and VD-A displayed lower nitrogen adsorption capacities in the medium-pressure region compared to SCD-A. Despite the comparable pore size distributions observed among these three samples (Table 1, BJH), density measurements (Figure 2b) indicated a clear trend: VD-A > SCD-A > FD-A. Structural analysis, combined with Figure 3, indicates that under the same volume conditions, FD-A and VD-A contain fewer mesopores than SCD-A. Specifically, VD-A’s adsorption curve in this region appeared more gradual, reflecting a reduction in the uniformity of its mesoporous structure. Remarkably, ND-A showed a substantial decrease in the nitrogen adsorption capacity in the medium-pressure region compared to SCD-A. A comprehensive analysis of pore size, density, and SEM images confirmed that under identical volume conditions, ND-A had significantly fewer mesopores.

In the high-pressure region, nitrogen undergoes macroscopic liquid-phase filling in macropores, demonstrating that both the aerogels and balsa wood possess macroporous structures. The specific surface areas of the wood aerogels are presented in Table 1. Combined with SEM analysis, the nitrogen adsorption–desorption curve aligns well with the observed structural characteristics. Compared to balsa wood, SCD-A exhibits a significantly higher specific surface area due to the formation of new pore structures following the removal of lignin and hemicellulose. Supercritical CO_2_ drying eliminates surface tension, preserving these pore structures intact during the drying process. Although the removal of hemicellulose and lignin increases both the number and volume of pores in wood aerogel, FD-A displays a smaller specific surface area than SCD-A. This is attributed to ice crystal formation during freeze-drying, which increases pore volume and reduces density. For the same wood aerogel volume, the reduced number of pores leads to a lower specific surface area, highlighting the critical role of pore quantity in determining the specific surface area of wood aerogels. Vacuum drying reduces the volume of wood aerogel, yet VD-A’s specific surface area remains comparable to that of SCD-A. This suggests that an increased number of pores can compensate for the loss in specific surface area caused by pore volume shrinkage. During the oven-drying process, stronger capillary forces compared to vacuum drying result in greater volume shrinkage and pore size reduction. However, the pore structure does not completely collapse, as confirmed by SEM observations. The OD-A sample exhibits higher nitrogen adsorption capacity in both the low-pressure and mid-pressure regions, indicating a well-developed pore structure. Within the same volume, OD-A exhibits a further increase in pore numbers without pore collapse, resulting in an additional increase in specific surface area. The specific surface area of ND-A is similar to that of balsa wood, as the presence of surface tension during drying causes some of the pore structures to disappear under capillary forces. A comparative analysis of the specific surface areas of VD-A, OD-A, and ND-A, along with Figure 3, shows that liquid surface tension can effectively regulate the pore size of wood aerogels. The pore structure disappears only when the surface tension reaches a critical threshold. By adjusting the temperature and environmental pressure, the surface tension can be precisely controlled to tailor the pore structure of wood aerogels.

### 3.7. Wood Aerogel Pore Size Analysis

**Figure 7:**
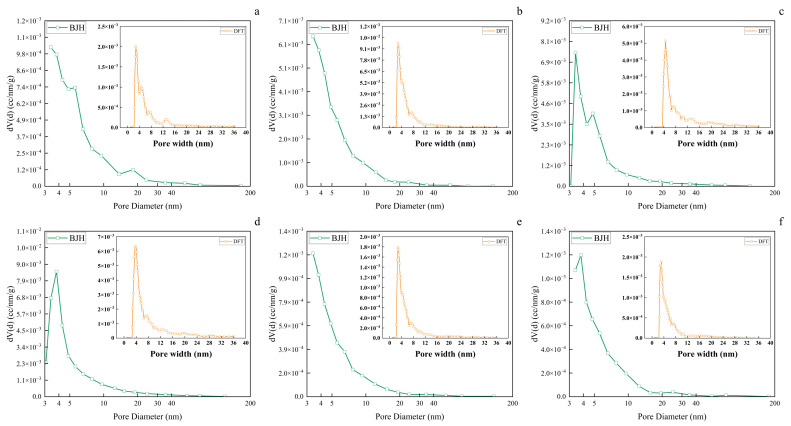
BJH desorption and DFT pore size distribution of wood aerogels and balsa wood. (**a**) Freeze-dried wood aerogel (FD-A). (**b**) Supercritical CO_2_-dried wood aerogel (SCD-A). (**c**) Vacuum-dried wood aerogel (VD-A). (**d**) Oven-dried wood aerogel (OD-A). (**e**) Naturally dried wood aerogel (ND-A). (**f**) Balsa wood (WB).

From Figure 7, it can be observed that the pore size of both balsa wood and wood aerogels are predominantly mesoporous. The BJH (desorption) and DFT average pore sizes of balsa wood and the wood aerogel are shown in Table 1. Compared to balsa wood, the peak position of the BJH desorption curve for SCD-A shifts toward smaller pore diameters, accompanied by a significant enhancement in mesoporous nitrogen adsorption capacity. Concurrently, DFT measurements demonstrate a synchronous enhancement in nitrogen adsorption. A comprehensive analysis of Figure 6 reveals a marked increase in the number of mesopores within the small-scale range in the SCD-A material. Analysis of the pore sizes of balsa wood and the average pore size of SCD-A reveals that the removal of hemicellulose and lignin alters the pore structure of the aerogel, leading to a reduction in the average mesopore size. Combined with SEM analysis, it indicates that the removal of hemicellulose and lignin introduces more smaller-sized mesopores into the wood aerogel. Compared with the SCD-A sample, FD-A exhibits a significant improvement in the mesoporous nitrogen adsorption capacity, with distinct characteristic peaks appearing in both the 5–10 nm and 10–20 nm ranges (Figure 7a BJH). It is noteworthy that although DFT testing detects new peaks within the 4–16 nm range, the nitrogen adsorption capacity of small-scale mesopores shows a clear decreasing trend. Data from Table 1 indicates that FD-A has a reduced specific surface area compared to SCD-A. A comprehensive analysis of Figure 6 reveals that the ice crystals formed during the freeze-drying process exert dual effects on the mesoporous structure of the wood aerogel: not only do they significantly reduce the number density of small-sized mesopores, but they also induce the formation of a new mesopore distribution pattern. In contrast to SCD-A, VD-A exhibits a slight increase in nitrogen adsorption capacity within the mesoporous range, along with the emergence of a new characteristic peak in the 5–10 nm region. However, the DFT-calculated nitrogen adsorption capacity significantly decreases. This phenomenon indicates a notable reduction in smaller-sized mesopores in the VD-A material, while the pore size distribution demonstrates a tendency to concentrate within the 5–10 nm range. VD-A exhibits an increase in both average pore size and density compared to SCD-A, with a clear increase in the average mesopore size. This demonstrates a significant influence of capillary forces on the pore structure of wood aerogel, where capillary action during drying reduces the number of small-scale mesopores, ultimately increasing the average mesopore size.

Compared with SCD-A, OD-A shows an increase in nitrogen adsorption capacity within the mesoporous range (Figure 7d BJH), while its nitrogen adsorption capacity for small-scale mesopores only exhibits a slight rise relative to VD-A (Figure 7d DFT). This indicates that OD-A has a higher total number of mesopores than SCD-A but a significantly lower number of small-scale mesopores. Compared to VD-A, OD-A experiences greater capillary forces during drying, leading to more pronounced volume shrinkage and an increase in the average mesopore size. This observation suggests that stronger capillary forces cause more small-scale mesopores to disappear. The nitrogen adsorption capacity of both mesopores and small-scale mesopores in ND-A is lower than that in OD-A and shows significant differences compared to SCD-A. This indicates a reduction in mesopore quantity during drying, with a substantial loss of small-scale mesopores. ND-A undergoes even greater capillary forces than OD-A, resulting in a decrease in both specific surface area and average pore size. This implies that more pore structures disappear during drying. As shown in Table 1, the variations in specific surface area and average pore size of SCD-A, VD-A, OD-A, and ND-A demonstrate that during the drying process, with increasing capillary forces, the aerogel volume shrinks while mesopore quantity initially increases; when capillary forces reach a critical intensity, more mesopores disappear, leading to reduced quantity along with decreased specific surface area and pore size.

### 3.8. Wood Aerogel Cyclic Water Absorption Performance

**Figure 8:**
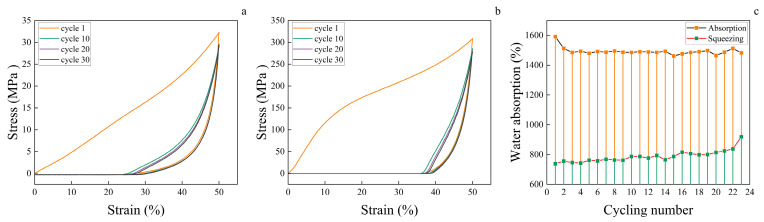
(**a**) The cyclic compression stress–strain curves of FD-A. (**b**) The cyclic compression stress–strain curves of SCD-A. (**c**) The cyclic water absorption performance curve of FD-A.

The strain in FD-A and SCD-A increases with the number of compression cycles, indicating damage to the pore structure during compression. After 30 compression cycles, the rebound height of FD-A was 13 mm (Figure 8a), significantly higher than that of SCD-A, which was 9 mm (Figure 8b). This difference mainly results from the effect of drying methods on the aerogel’s pore structure. During freezing, ice crystal formation compresses cellulose fibers in the aerogel, leading to an elastic structure [28]. This elastic structure helps the aerogel resist plastic deformation, ultimately giving FD-A a lower residual strain. FD-A exhibits cyclic water absorption capability, achieving up to 23 absorption cycles, as shown in Figure 8c. As depicted in Figure 2c, the water absorption rate of FD-A diminishes with increasing cycles. This decline is attributed to the collapse of the pore structure during compression, which ultimately reduces the water absorption capacity of FD-A. The SCD-A sample exhibits low compression resilience and poor water absorption performance. Experimental results indicate that the sample undergoes structural failure during the first compression cycle in the cyclic water absorption test and thus completely lacks the capability for cyclic water absorption. VD-A, OD-A, and ND-A show significant pore shrinkage during drying (Figure 3), higher density (Figure 2b), and lower water absorption (Figure 4a). These samples cannot withstand compression and therefore do not possess cyclic water absorption performance.

## 4. Conclusions

In this study, wood aerogels were prepared through NaClO_2_ and NaOH solution treatments using five drying methods: freeze-drying, supercritical CO_2_ drying, vacuum drying, oven drying, and natural drying. Among these, freeze-drying required the longest duration (22 h), whereas vacuum drying was the most time-efficient (3 h). The density of the wood aerogels was significantly influenced by the drying method. Freeze-dried aerogels exhibited the lowest density, coupled with the highest water absorption rate and capacity. In contrast, naturally dried wood aerogels showed the opposite trend, with the highest density but poorest absorption performance. Notably, supercritical CO_2_ wood aerogels demonstrated exceptional thermal insulation properties, while naturally dried wood aerogels exhibited superior thermal conductivity. Structural characterization revealed that the balsa wood aerogels were predominantly mesoporous. The performance characteristics of these wood aerogels were fundamentally governed by their pore structures, which were directly determined by the respective drying processes.

## Figures and Tables

**Table 1:** Specific surface area and pore size of wood aerogel and balsa wood.

Drying Method	FD-A	SCD-A	VD-A	OD-A	ND-A	BW
Specific Surface Area m^2^/g	4.4	5.4	5.3	14.3	1.2	1.1
Pore width (HK) nm	1.1	0.4	0.4	0.4	1.6	1.9
Pore width (BJH) nm	3.4	3.4	3.4	3.8	3.4	3.8
Pore width (DFT) nm	2.8	2.8	4.5	3.8	2.8	3.2

## Data Availability

Data are contained within the article.
